# Novel Algorithm to Estimate Fat‐Free Muscle Volumes in Women Using the Urinary Deuterated‐Creatine Dilution Method

**DOI:** 10.1002/jcsm.13872

**Published:** 2025-07-09

**Authors:** Darren Yuen Zhang Tan, Wei Fun Cheong, Shanshan Ji, Amaury Cazenave‐Gassiot, Jane Cauley, Liang Shen, Eu‐Leong Yong

**Affiliations:** ^1^ Department of Obstetrics and Gynecology, Yong Loo Lin School of Medicine National University of Singapore Singapore Republic of Singapore; ^2^ Singapore Lipidomics Incubator, Life Sciences Institute National University of Singapore Singapore Republic of Singapore; ^3^ Department of Biochemistry, Yong Loo Lin School of Medicine National University of Singapore Singapore Republic of Singapore; ^4^ Department of Epidemiology, Graduate School of Public Health University of Pittsburgh Pittsburgh Pennsylvania USA; ^5^ Biostatistics Unit, Yong Loo Lin School of Medicine National University of Singapore Singapore Republic of Singapore

**Keywords:** body composition, creatine, creatinine, MRI, muscle mass, sarcopenia

## Abstract

**Background:**

Muscle mass declines after menopause and is a key risk factor for frailty, falls and poor physical function as women age. The deuterated creatine (D_3_Cr) dilution method provides a direct assessment of muscle mass, but its accuracy in Asian women has not been evaluated. Our aim was to develop a new D_3_Cr algorithm incorporating anthropomorphic variables that can estimate fat‐free muscle mass, using magnetic resonance imaging (MRI) as the reference standard.

**Methods:**

The Integrated Women's Health Programme (IWHP) enrolled 1201 healthy community‐dwelling women, aged 45–69 years at baseline, who attended gynaecological clinics from 2014 to 2016. Between February 2021 and July 2023, 894 participants were recontacted, and 451 of the respondents agreed to ingest 30 mg of D_3_Cr and had available MRI measurements of fat‐free thigh and erector spinae volumes. Urinary levels of creatine, creatinine and D_3_‐creatinine levels were measured by tandem mass spectrometry 4 days after ingestion of D_3_Cr. Muscle mass was estimated using the two D_3_Cr algorithms (D_3_Cr_original_ and D_3_Cr_modified_) in current use and a newly developed algorithm (D_3_Cr_Ht‐Wt_) incorporating anthropometric variables that estimate fat‐free muscle volumes. Pearson's correlation analyses were used to compare the performances of the D_3_Cr algorithms with MRI. Bland–Altman analysis was used to ascertain agreement between D_3_Cr_Ht‐Wt_ and MRI.

**Results:**

Participants (*n* = 451, mean age 62.6 ± 5.9) were randomly divided into training (*n* = 367) and validation (*n* = 84) cohorts. In the training cohort, stepwise multivariable regression modelling indicated that age (*β* = −0.011, *p* = 0.076) and ethnicity (*β* = 0.154, *p* = 0.317 [Indian]; *β* = −0.012, *p* = 0.942 [Malay] compared to Chinese) were not associated with fat‐free muscle volumes. In the final model, D_3_Cr‐determined creatine pool size (*β* = 0.032, *p* < 0.001), body weight (*β* = 0.030, *p* < 0.001) and height (*β* = 4.336, *p* < 0.001) were independently associated with fat‐free muscle volumes and were incorporated into a new algorithm (D_3_Cr_Ht‐Wt_). In a separate validation cohort, muscle volumes estimated using the D_3_Cr_Ht‐Wt_ algorithm (*R* = 0.813) had a higher correlation with MRI‐measured fat‐free muscle volumes than both D_3_Cr_original_ (*R* = 0.672) and D_3_Cr_modified_ (*R* = 0.692) algorithms. Bland–Altman analysis indicated a low bias of 0.112 L and limits of agreement of −0.969 L to +1.190 L.

**Conclusions:**

Muscle volumes estimated with the D_3_Cr_Ht‐Wt_ algorithm had high correlation and agreement with MRI‐measured fat‐free muscle volumes. The convenience of the D_3_Cr method for participants suggests its potential to be a clinically relevant method for assessing fat‐free muscle volumes in sarcopenia studies.

## 1 Introduction

Skeletal muscle is one of the largest body compartments and has a fundamental influence on health [1]. Muscle mass declines after menopause and is a key risk factor for frailty, falls and poor physical function as women age [2]. Despite muscle mass being an essential component in the definition for sarcopenia [3, 4], the degree to which reduction of muscle mass contributes to loss of functional capacity and risk of disability has not been established [5]. Muscle mass or volumes can be quantified directly with 3D imaging modalities such as magnetic resonance imaging (MRI) [6, 7]. However, MRI methods require highly trained staff, expensive imaging equipment and arduous manual delineation of different muscle groups, rendering this tool unsuitable for large community studies [7, 8]. Dual energy X‐ray absorptiometry (DXA) measurement of appendicular lean mass (ALM) has been advocated as a reference standard due to the method's ease of use, reduced cost, low radiation and accessibility [9]. However, DXA‐ALM includes non‐muscle elements such as skin and connective tissue [9] and is unable to delineate fat infiltration of muscle. Bioelectric impedance analysis (BIA) is safe, inexpensive, portable and easy to use. However, BIA does not measure muscle mass directly and is sensitive to hydration status, recent activity and time spent lying horizontal and have large individual prediction errors [9]. Several cross‐sectional and longitudinal ageing cohort studies have reported little or no relationship between low lean body mass measured by the DXA and BIA with health‐related outcomes such as functional capacity, disability and mortality [5].

The deuterated creatine (D_3_Cr) dilution method is a promising method to measure total skeletal muscle mass in the community as it requires only a few drops of urine [10, 11, 12]. The D_3_Cr method exploits the fundamental role that creatine has in ATP–ADP phosphocreatine energy transfer in muscle cells [13]. The D_3_Cr method estimates creatine pool size as a metric of total skeletal muscle mass. Subjects ingest a small oral dose of D_3_Cr, which is mostly absorbed and incorporated into skeletal muscles within days [10]. D_3_Cr is converted into labelled creatinine at a constant rate and excreted in the urine [10, 14]. The creatine pool size can be estimated by measuring deuterated creatinine enrichment (as a portion of endogenous unlabelled creatinine) in urine with liquid chromatography–tandem mass spectrometry (LC–MS/MS) [10, 13]. Emergent studies indicate associations of D_3_Cr‐measured muscle mass with falls and fractures, mobility, disability and mortality [15, 16].

Although strong correlations exist between D_3_Cr‐ and DXA‐ or MRI‐estimated muscle mass in men, less consistent associations were observed in women [16, 17, 18]. In order to improve the estimation of total creatine pool size, a spillage correction algorithm was devised to account for small doses of D_3_Cr that was directly excreted in the urine following oral ingestion [11]. This estimation of creatine spillage was problematic as the algorithm was empirically deduced from a limited number of observations. Furthermore, women spill more of the ingested D_3_Cr dose than men [10, 11], leading to inaccurate estimations of muscle mass in women. There is a need for an improved algorithm for D_3_Cr‐measured muscle mass in women that does not rely on spillage correction. We hypothesize that since ethnicity, age, height and/or weight were significantly correlated with muscle mass [19], their incorporation into a D_3_Cr algorithm, alone or in combination, would result in a new algorithm that avoids the need for a spillage correction.

Recently, a specialized MRI protocol requiring less than 10 min, coupled with automated estimation of fat‐free thigh muscle volumes, has been reported in large‐scale studies to correlate with key sarcopenia functional outcomes including hospital night stays, handgrip strength, stair climbing ability, walking pace, falls and all‐cause mortality [20, 21]. As common modalities such as DXA cannot measure detailed muscle composition, the ability of the MRI protocol to measure fat‐free muscle volumes allows for clearer associations between muscle volumes and important health outcomes [22]. It would be particularly advantageous if a new D_3_Cr algorithm for women can also estimate fat‐free muscle volumes.

The overall objective is to develop an improved D_3_Cr algorithm that correlated with fat‐free muscle volumes in midlife women. We split our data into training and validation cohorts. In the training cohort, we aimed to develop a new D_3_Cr algorithm, utilizing regression modelling to examine variables such as age, ethnicity and anthropometric measurements, with MRI‐measured fat‐free muscle volumes as the reference standard. Finally, in the validation cohort, we compared the abilities of the newly developed D_3_Cr algorithm and existing algorithms to estimate MRI‐measured fat‐free muscle volumes.

## 2 Methods

### 2.1 Study Design

**FIGURE 1:**
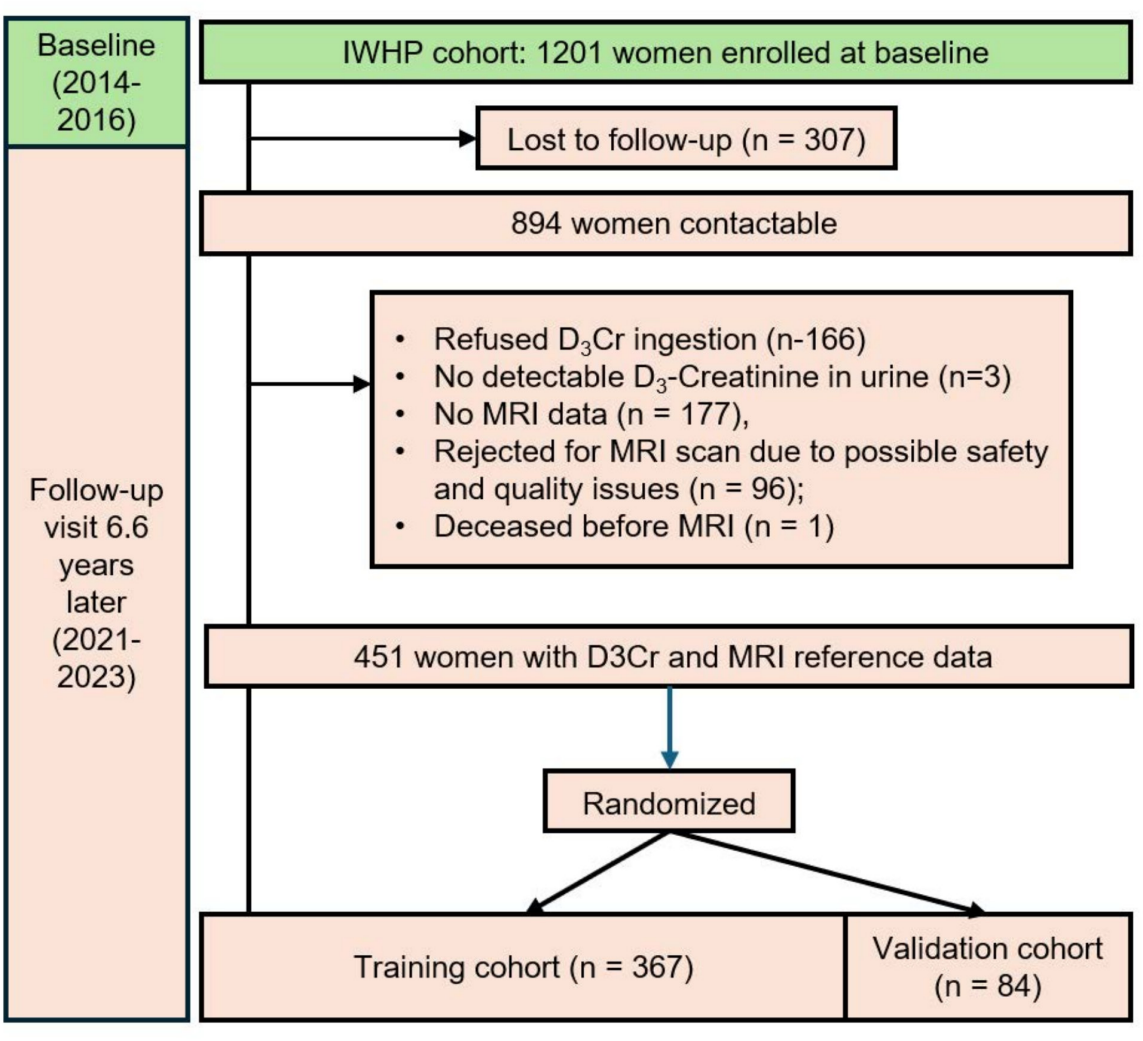
Participant flow for the D_3_Cr substudy of the Integrated Women's Health Programme longitudinal cohort.

Participants were from the Integrated Women's Health Programme (IWHP), a prospective cohort study on health issues faced by midlife Singaporean women. Details of the IWHP cohort had been previously described [23]. The participant flow chart is shown in Figure 1. In brief, 1201 community‐dwelling women, aged 45–69 years at baseline, who attended gynaecological clinics for routine health screening were enrolled into the IWHP longitudinal cohort from 2014 to 2016. Exclusion criteria included potentially life‐threatening diseases, pregnancy or low literacy. Participants were recontacted between February 2021 and July 2023, and 894 (74.4%) contactable respondents were invited to participate in this D_3_Cr substudy. Of these 894 participants, 451 agreed to ingest 30 mg of D_3_Cr and had available MRI data for analysis. Participants excluded from this analysis included those who refused D_3_Cr ingestion (*n* = 166), did not have detectable levels of urinary D_3_‐creatinine (*n* = 3), did not have MRI data (*n* = 177), were rejected from MRI scan due to possible safety and quality issues (*n* = 96) or were deceased before the MRI scan (*n* = 1). The median (IQR) time gap between the MRI and the D_3_Cr measurements was 56 (0–317) days. Both the IWHP and D_3_Cr studies were approved by the Domain Specific Review Board of the National Healthcare Group, Singapore (Reference Numbers: 2020/00201 and 2021/00076). All participants gave written informed consent for both studies.

### 2.2 Height, Weight and Handgrip Strength Measurements

Height was measured twice and averaged, and weight was measured once using the SECA 769 Electronic Measuring Station (Seca, Hamburg, Germany). Body mass index (BMI) was calculated as weight divided by height squared (kg/m^2^). Handgrip strength was assessed with a dynamometer (Jamar, Bolingbrook, IL). Two measurements were taken for each arm, and the maximum value of the four readings was analysed.

### 2.3 Reference Standard: Fat‐Free Thigh Plus Erector Spinae Muscle Volumes by MRI

MRI scans were performed in a Siemens Biograph mMR 3‐T MRI scanner (Siemens Healthineers, Erlangen Germany) using a 6‐min neck‐to‐knee two‐point Dixon VIBE protocol by trained radiographers [24]. Automatic image analysis with manual quality control was performed using AMRA Researcher (AMRA Medical AB, Linköping, Sweden). The image acquisition and post‐processing protocols have been described elsewhere [24]. In brief, water and fat‐separated volumetric data from the neck to the knee were obtained, the images were calibrated using fat‐referenced MRI and a nonrigid registration of multiple atlases was applied to automatically segment the musculature [25]. After quality control, fat‐free muscle volumes, defined as the volume of all voxels in the mask with less than 50% fat concentration, were measured [25].

### 2.4 D_3_Cr Dilution Assay

#### 2.4.1 Urine Sample Collection

On the first visit, participants ingested a single 30‐mg dose of D_3_Cr. Participants were not restricted to any specific diet or lifestyle. On a second visit, approximately 96 h after the first visit, participants were asked to provide a fasted urine sample. Isotopic steady state for urinary D_3_‐creatinine enrichment has been reported to be achieved 3–5 days after ingestion of oral D_3_‐creatine [10]. It is relevant to note that some women require a longer period of up to 76 h to achieve steady state [10]. Since the enrichment ratio remained at steady state for up to 120 h, we selected 96 h to capture steady‐state D_3_‐creatinine enrichment following established protocols [11, 16]. Urine samples were processed within 30 min by centrifuging at 3000 *g* for 10 min at 4°C to precipitate debris and insoluble materials. The supernatant was aliquoted and stored at −80°C until analysis.

#### 2.4.2 Chemicals and Reagents

Deuterated creatine with ≥ 97% chemical purity was purchased from Cambridge Isotope Laboratories Inc. (Tewksbury, MA, USA) and encapsulated at the Greenpark Compounding Pharmacy (Houston, TX, USA). Deuterated standards such as D_5_‐creatine (D_5_Cr), D_5_‐creatinine (D_5_Crn) and D_3_‐creatinine (D_3_Crn) were purchased from CDN Isotopes Inc. (Quebec, Canada). Creatine, creatinine and formic acid standards were purchased from Sigma‐Aldrich (St. Louis, MO, USA).

#### 2.4.3 LC–MS/MS Methods

LC–MS/MS analyses were performed on an Agilent 1290 Infinity liquid chromatography system coupled with an Agilent 6490 Triple Quadrupole (iFunnel) mass spectrometer with an Agilent Jet Stream (AJS)‐ESI source (Agilent Corp., Santa Clara, CA, USA). Multiple reaction monitoring (MRM) transitions were obtained and optimized using the commercial standards of Crn, Cr, D_3_Crn, D_5_Crn and D_5_Cr using an established protocol [26]. Product ion scans were performed to determine the MRM transition of target analytes by selecting the most distinctive and abundant product ion yield and the respective optimal collision energy (V) (Figure S1). The linear range of detection for individual standards was determined via the optimized LC‐MRM method using a pooled control urine extract (from individuals without D_3_Cr ingestion), spiked with respective standards ranging from 0.1 to 10 000 ng/mL (Table S1). The assessment of mean equivalence of D_3_Crn versus Crn shows high comparability, with a mean percentage difference of < 0.2% (Table S2). Due to a much higher concentration of unlabelled endogenous Crn in urine samples as compared to the amount of D_3_Crn (approximately 1000‐fold difference in intensities), a less intense ion transition (M + 2) for Crn was used in the LC‐MRM analysis [26]. Reduced intensity of Crn peak areas allows for measurement of both Crn and D_3_Crn in the same sample concurrently. Response ratios of M + 2 Crn and M + 0 Crn were determined experimentally using two different concentrations of Crn solutions to account for response variations on different LC–MS systems [26]. The mean of all response ratio measurements was 0.002469 with an RSD of ± 2.7% (Table S3) and was subsequently used as the correction factor in quantitation of Crn in urine samples. Validation of the corrected Crn peak area was conducted by comparing it with its equivalence counterpart, D_3_Crn. The mean percentage difference between corrected Crn and D_3_Crn was < 3% (Table S4). For quality control, the precision and bias for within‐run and between‐run assays were obtained from five independent analyses of varying levels of reference samples, prepared using spiked standards of 50, 500, 3000 and 5000 ng/mL in pooled control urine extracts. Precision of the average response ratio yielded was RSD < 3.0%, and the mean percentage difference of the corrected Crn peak area to D_3_Crn peak area was < 2.5%. Precision and bias of all measured analytes, at different concentration levels, were < 15%, with an overall between‐run precision of < 8% (Table S5).

#### 2.4.4 Sample Preparation and LC‐MRM Analyses

Urine samples were divided into batches of 40. Sample preparation, extraction of metabolites and LC–MS/MS analysis were performed one batch at a time. Quality control samples, namely, technical quality control (TQC), sample processing quality control (PQC) and column conditioning (CC), prepared from pooled samples of urine from participants, were inserted into every batch. TQC and PQC samples were used to evaluate batch‐to‐batch sample preparation and analytical precision.

The sample extraction procedure was adapted and modified from Shankaran and colleagues [11]. In brief, QCs and urine samples were thawed, vortexed and centrifuged at 21 100 *g* for 10 min at 4°C. Urine (100 μL) was transferred into a microfuge tube, and 50 μL of internal standard solution, consisting of D_5_Crn and D_5_Cr, was spiked into the urine samples followed by the addition of 250‐μL methanol as the extraction solvent. Methanol was used as an extraction medium, in place of acetonitrile, for a more reproducible signal‐to‐noise ratio. Final concentrations of D_5_Crn and D_5_Cr in the extraction mixture were 250 and 2500 ng/mL, respectively. The mixture was vortexed and incubated at 4°C for 1 h. After incubation, extraction was carried out by vortexing the mixture vigorously for 2 min. Metabolites and precipitated proteins were then separated by centrifuging at 21 100 *g* for 10 min at 4°C. The supernatant was retrieved, and solid‐phase extraction (SPE) was used to remove interfering compounds in the urine extract. Prior to SPE, the sample was diluted 50× with 70% acetonitrile and then loaded into an SPE cartridge that was pre‐equilibrated with methanol. Flowthrough was collected after 10 min by gravity elution or with low pressure as needed and centrifuged at 21 100 *g* for 10 min at 4°C before being transferred into an LC–MS vial.

Two microliters of sample was injected for chromatographic separation in a Cogent Diamond Hydride column (4 μm, 4.6 mm × 100 mm) (Cogent, Leland, NC, USA). The column temperature was maintained at 25°C throughout the analysis. Each sample was injected twice for replicate analysis. An Agilent InfinityLab Quick Change inline filter (4.6 mm × 0.5 μm) was installed between the autosampler and column to remove particulate contaminants from samples. Prepared before each batch of analysis, the mobile phase consisted of Solvent A (ultrapure water with 0.05% formic acid, pH 3) and Solvent B (acetonitrile with 0.05% formic acid, pH 3). The flow rate was kept at 0.8 mL/min. The gradient elution started at 70% B, decreased to 40% at 1 min and then to 5% at 4 min. It was maintained at 5% B until 6 min before being returned to 70% B at 8 min. The optimum operating parameters of the AJS‐ESI interface in both positive and negative modes were as follows: delta EMV, +200 and −200 V; gas temperature, 250°C; gas flow, 14 L/min; nebulizer, 45 psi; sheath gas temperature, 320°C; sheath gas flow, 11 L/min; capillary, +3000 and −3000 V; nozzle voltage, +1500 and −1500 V; iFunnel high‐pressure RF, 150 V; and low‐pressure RF, 60 V. Nitrogen gas was used as the curtain and collision gas. All data were recorded and processed by using Agilent MassHunter B07.00 (Agilent Corp., Santa Clara, CA, USA). As maintenance, the LC–MS source was cleaned between each batch of analysis, where the spray shield, capillary cap and spray nebulizer were removed and sonicated in 50% isopropanol and rinsed with methanol. Mass calibration was performed thereafter to maintain high accuracy.

### 2.5 Original D_3_Cr Algorithm for Total Skeletal Muscle Mass With Spillage Correction (D_3_Cr_original_)

To our knowledge, most muscle mass studies utilizing the deuterated creatine method use the original spillage correction algorithm developed by Shankaran et al. [11, 27] to estimate muscle mass (D_3_Cr_original_). Endogenous Cr and Crn, as well as the deuterated metabolite D_3_Crn, were measured using LC–MS/MS as described above. The following equations were used to calculate D_3_Cr_original_ [11]:

- Spillage correction (mg) = (exp(1.2913 × ln(Cr/Crn ratio) + 0.7783) × 30 mg (D_3_Cr dose)
- Cr pool size (g) = (0.03[g] − ((spillage correction [mg]/1000) × (131.1/134.1)))/enrichment ratio, where enrichment ratio = D_3_Crn/ (D_3_Crn + Crn)
- Total body muscle mass estimated by D_3_Cr_original_ (kg) = Cr pool size [g]/4.3 [g/kg]

### 2.6 D_3_Cr Algorithm for Total Skeletal Muscle With Modified Spillage Correction (D_3_Cr_modified_)

A modified spillage algorithm for the D_3_Cr method has been recently published [28]. In this modified D_3_Cr method, a three‐tier spillage correction was applied depending on endogenous Cr/Crn ratios in urine [28].

- For a Cr/Crn ratio of < 0.015, no spillage correction is applied.
- For a Cr/Crn ratio of 0.015–0.15, spillage correction (mg) = (exp((0.9424 × ln(Cr/Crn ratio)) − 0.1314)) × 30.
- For a Cr/Crn ratio of > 0.15, spillage correction (mg) = (exp((1.6246 × ln(Cr/Crn ratio)) − 1.895)) × 30.

### 2.7 Developing a D_3_Cr Algorithm (*Without* Spillage Correction) to Estimate MRI‐Measured Fat‐Free Thigh and Erector Spinae Muscle Volumes

To develop a new algorithm for the urinary D_3_Cr dilution assay, we firstly examined the associations of ethnicity, age, height and weight with MRI‐measured fat‐free thigh and erector spinae muscle volumes using linear regression. BMI was left out of the model as height and weight comparatively provided better insights of the influence of body proportion [19]. Urine samples were randomly split into training (80%) and validation (20%) sets. Allocation of 20% of the dataset as a validation set and the remaining 80% as the training set has been reported to yield the best results for model development [29].

Linear regression was performed in the training set to assess the association of individual variables, including ethnicity, age, height and weight, with muscle volumes. Based on the resulting regression equations, variables were selected to develop a new algorithm that best estimated MRI‐measured thigh and erector spinae.

Since simple anthropomorphic measures alone can estimate muscle mass to a certain extent [19], we also performed regression analyses to generate a separate algorithm to estimate muscle volumes using height and weight only (MV_Ht‐Wt_).

Pearson's correlation coefficients were used to compare MRI‐measured thigh and erector spinae muscle volumes with those estimated by the various algorithms. Analyses were performed using SPSS 29.0. Pearson's chi‐square analysis was used for categorical variables, and independent‐samples *t*‐test was used for continuous variables to assess differences between the training and validation sets. Bland–Altman analysis was used to assess the agreement between the new algorithm and MRI‐measured thigh and erector spinae muscle volumes. Pearson's correlation analysis was also used to compare the performance of each algorithm with handgrip strength. All statistical tests are two‐sided, and significance was indicated by a two‐sided *p* value of < 0.05. Figures were generated in RStudio (2024.12.1, Build 563) using R‐4.4.3 and packages readxl, dplyr, ggplot2, viridis, sfsmisc, ggpubr, grid and BlandAltmanLeh.

## 3 Results

### 3.1 Participant Characteristics

Participants (*n* = 451) with complete data on both urinary D_3_Cr‐estimated and MRI‐measured muscle volumes were analysed (Figure 1). The characteristics of participants who were re‐contactable (*n* = 894) are shown in Table S6. Participants who were excluded from the analysis were more likely to be of lower education levels. No other significant differences were observed. The analytic group had a mean age of 62.6 ± 5.9, were mostly of Chinese ethnicity (85.2%), were postmenopausal (96.2%) and had a mean BMI of 24.6 ± 4.4 kg/m^2^ (Table 1).

**TABLE 1:** Characteristics of participants in the training and validation datasets. Continuous variables are reported as mean ± SD and categorical variables are reported as *n* (%).

	All participants (*n* = 451)	Training set (*n* = 367)	Validation set (*n* = 84)	*p*
Age	62.6 ± 5.9	62.6 ± 5.9	62.8 ± 6.3	0.781
Ethnicity				0.314
Chinese	368 (81.6)	299 (81.2)	69 (18.8)	
Malay	27 (6.0)	24 (88.9)	3 (11.1)	
Indian	37 (8.2)	27 (73.0)	10 (27.0)	
Other	19 (4.2)	17 (89.5)	2 (10.5)	
Education				0.419
No formal/primary	43 (9.6)	38 (88.4)	5 (11.6)	
Secondary and pre‐university	286 (64.0)	230 (80.4)	56 (19.6)	
University	118 (26.4)	98 (83.1)	20 (16.9)	
Monthly household income				0.273
< $3000	92 (25.3)	77 (83.7)	15 (16.3)	
$3000–$6999	121 (33.3)	92 (76.0)	29 (24.0)	
≥ $7000	150 (41.3)	124 (82.7)	26 (17.3)	
Marital status				0.583
Not married	107 (23.7)	89 (83.2)	18 (16.8)	
Married	344 (76.3)	278 (80.8)	66 (19.2)	
Employment status				0.924
Unemployed	190 (42.1)	155 (81.6)	35 (18.4)	
Employed	261 (57.9)	212 (81.2)	49 (18.8)	
Menopausal status				0.137
Pre‐menopausal	6 (1.3)	6 (100.0)	0 (0.0)	
Peri‐menopausal	11 (2.5)	11 (100.0)	0 (0.0)	
Post‐menopausal	429 (96.2)	82 (19.1)	347 (80.9)	
Height (m)	1.56 ± 0.06	1.56 ± 0.06	1.56 ± 0.05	0.899
Weight (kg)	59.5 ± 10.8	59.7 ± 10.8	58.8 ± 10.8	0.494
BMI (kg/m^2^)	24.6 ± 4.4	24.6 ± 4.4	24.2 ± 4.2	0.463
Thigh and spinae erector muscle volume (L)	7.01 ± 1.01	7.00 ± 1.00	7.02 ± 0.95	0.881
D_3_Cr_original_ (kg)	16.2 ± 4.1	16.1 ± 4.3	16.5 ± 3.06	0.340
D_3_Cr_modified_ (kg)	17.7 ± 3.0	17.8 ± 3.1	17.2 ± 2.7	0.139
D_3_Cr_Ht‐Wt_ (L)	7.00 ± 0.78	7.00 ± 1.02	7.02 ± 0.95	0.232
MV_Ht‐Wt_ (L)	7.01 ± 0.67	7.01 ± 0.67	6.97 ± 0.68	0.574

### 3.2 Muscle Mass Estimated by Current D_3_Cr Algorithms Versus MRI‐Measured Muscle Volumes

**FIGURE 2:**
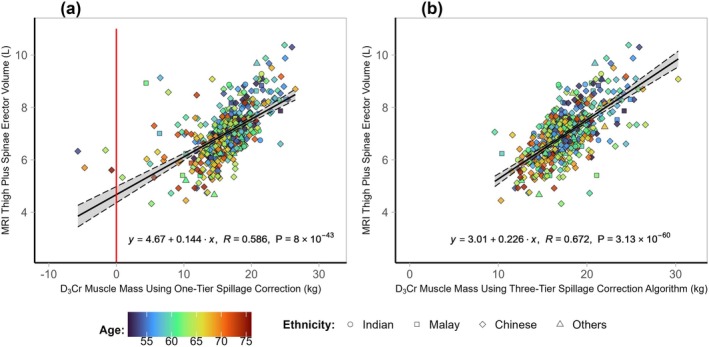
Evaluation of D_3_Cr algorithms in current use. Pearson correlations between (a) D_3_Cr_original_ and (b) D_3_Cr_modified_ algorithms and MRI‐measured thigh and erector spinae muscle volumes. Individuals (*n* = 451) in scatter plots are indicated by dots representing combined data from training and validation datasets. Regression lines and 95% CI are in grey. The red line marks zero muscle mass. Legend: circle = Indian, square = Malay, diamond = Chinese, triangle = others. The heat map indicates age: blue = youngest, red = oldest.

Muscle mass was estimated using the two D_3_Cr algorithms, D_3_Cr_original_ and D_3_Cr_modified_, in current use and compared to the sum of fat‐free thigh and erector spinae muscle volumes measured by MRI (reference standard). Scatter plots of total skeletal muscle mass estimated using D_3_Cr_original_ versus MRI‐measured thigh plus erector spinae muscle volumes are shown in Figure 2a. It is noteworthy that the D_3_Cr_original_ method gave rise to negative muscle mass (i.e., less than 0 kg) in four women (Figure 2a). Examination of these subjects revealed that negative muscle mass was due to high urine creatine/creatinine ratios, resulting in calculated urinary spillage that was higher than the ingested dose of deuterated creatine (data not shown). The correlation of the D_3_Cr_original_ with MRI‐measured fat‐free muscle volumes was moderate at *R* = 0.586. Use of the modified three‐tier spillage correction algorithm (D_3_Cr_modified_) resulted in a stronger correlation of *R* = 0.672 and the elimination of negative muscle mass (Figure 2b).

### 3.3 Development of a New D_3_Cr Algorithm Incorporating Height and Weight Without Spillage Correction (D_3_Cr_Ht‐Wt_) in the Training Cohort

Subjects were randomly split into training (*n* = 367) and validation (*n* = 84) cohorts (Figure 1). The training cohort was used to develop a new D_3_Cr algorithm (without spillage correction) with MRI‐measured fat‐free thigh plus erector spinae muscle volumes as the reference. Correlation analysis was conducted to determine the suitability of selected variables for model development. The age, height, weight, BMI and enrichment ratio were significantly correlated with MRI‐measured fat‐free thigh and erector spinae muscle volumes (Table S7). Linear regression using the stepwise modelling method was conducted to determine significant variables for inclusion into the new D_3_Cr algorithm (Table 2). Multicollinearity among the variables was assessed using the variance inflation factor, and all values were below the threshold of 3. In the training cohort, stepwise multivariable regression modelling indicated that age (*β* = −0.011, *p* = 0.076) and ethnicity (*β* = 0.154, *p* = 0.317 [Indian]; *β* = −0.012, *p* = 0.942 [Malay] compared to Chinese) were not associated with fat‐free muscle volumes, and these two variables were dropped from the final model (Table 2). In the final model, the D_3_Cr‐determined creatine pool size (*β* = 0.032, *p* < 0.001), body weight (*β* = 0.030, *p* < 0.001) and height (*β* = 4.336, *p* < 0.001) were independently associated with fat‐free muscle volumes and were incorporated into a new algorithm (D_3_Cr_Ht‐Wt_). We chose height and weight, instead of BMI, for inclusion into the model development equation because their correlation coefficients of *R* = 0.475 and 0.566, respectively, were higher than for BMI (*R* = 0.373) (Table S7). The creatine pool size, height and weight were independently associated with MRI‐measured thigh and erector spinae muscle volumes (Table 2), and their parameter estimates were incorporated into the following regression equation for estimating MRI‐measured thigh and erector spinae muscle volumes:

- Cr pool size = 0.03 [g]/enrichment ratio, where enrichment ratio = D_3_Crn/(D_3_Crn + Crn)
- Thigh and erector spinae muscle volumes estimated by D_3_Cr_Ht‐Wt_ (L) = 0.032 × creatine pool size + 0.030 × body weight [kg] + 4.336 × height [m] − 4.134

**TABLE 2:** Multivariable regression modelling to improve D_3_Cr prediction of MRI‐measured thigh and erector spinae muscle volumes without spillage correction. Variables examined are the creatine pool size (estimated by measuring D_3_‐creatinine enrichment [D_3_Crn as a portion of endogenous unlabelled creatinine] in urine), age, ethnicity, height and weight.

	Unadjusted		Model 1		Model 2		Model 3	
	*β*	*p*	*β*	*p*	*β*	*p*	*β*	*p*
Creatine pool size^a^	0.048	< 0.001	0.045	< 0.001	0.031	< 0.001	0.032	< 0.001
Age			−0.024	0.001	−0.011	0.076		
Ethnicity								
Indian			0.154	0.317				
Malay			−0.012	0.942				
Chinese	Reference							
Height					4.183	< 0.001	4.336	< 0.001
Weight					0.029	< 0.001	0.030	< 0.001
Constant	3.082	< 0.001	4.854	< 0.001	−3.112	0.006	−4.134	< 0.001
Adjusted *R* ^2^	0.426		0.438		0.578		0.576	

^a^ Creatine pool size was derived from the enrichment ratio: $\frac{0.03\;g\;\text{of}\;D_{3}\mathrm{Cr}\;\text{oral dose}}{\text{enrichment ratio}}$.

*Note:* Model 1: Creatine pool size, age and ethnicity. Model 2: Creatine pool size, age, height and weight. Model 3: Creatine pool size, height and weight.

### 3.4 Development of an Algorithm to Estimate Muscle Volume Incorporating Height and Weight Only (MV_Ht‐Wt_) in the Training Cohort

Studies have shown that algorithms using anthropometric variables alone can estimate muscle volumes fairly accurately [19]. We generated linear regression models incorporating only height and weight alone (MV_Ht‐Wt_) using MRI‐measured fat‐free thigh and erector spinae muscle volumes as the reference. In the training cohort, the following linear regression equation using only height and weight best estimated MRI‐measured fat‐free muscle volumes:

- Thigh and erector spinae muscle volumes estimated by MV_Ht‐Wt_ (L) = 0.045 × body weight [kg] + 6.266 × height [m] − 5.438

### 3.5 Estimation of MRI‐Measured Fat‐Free Muscle Volumes by D_3_Cr_Ht‐Wt_ and MV_Ht‐Wt_ in the Validation Cohort

**FIGURE 3:**
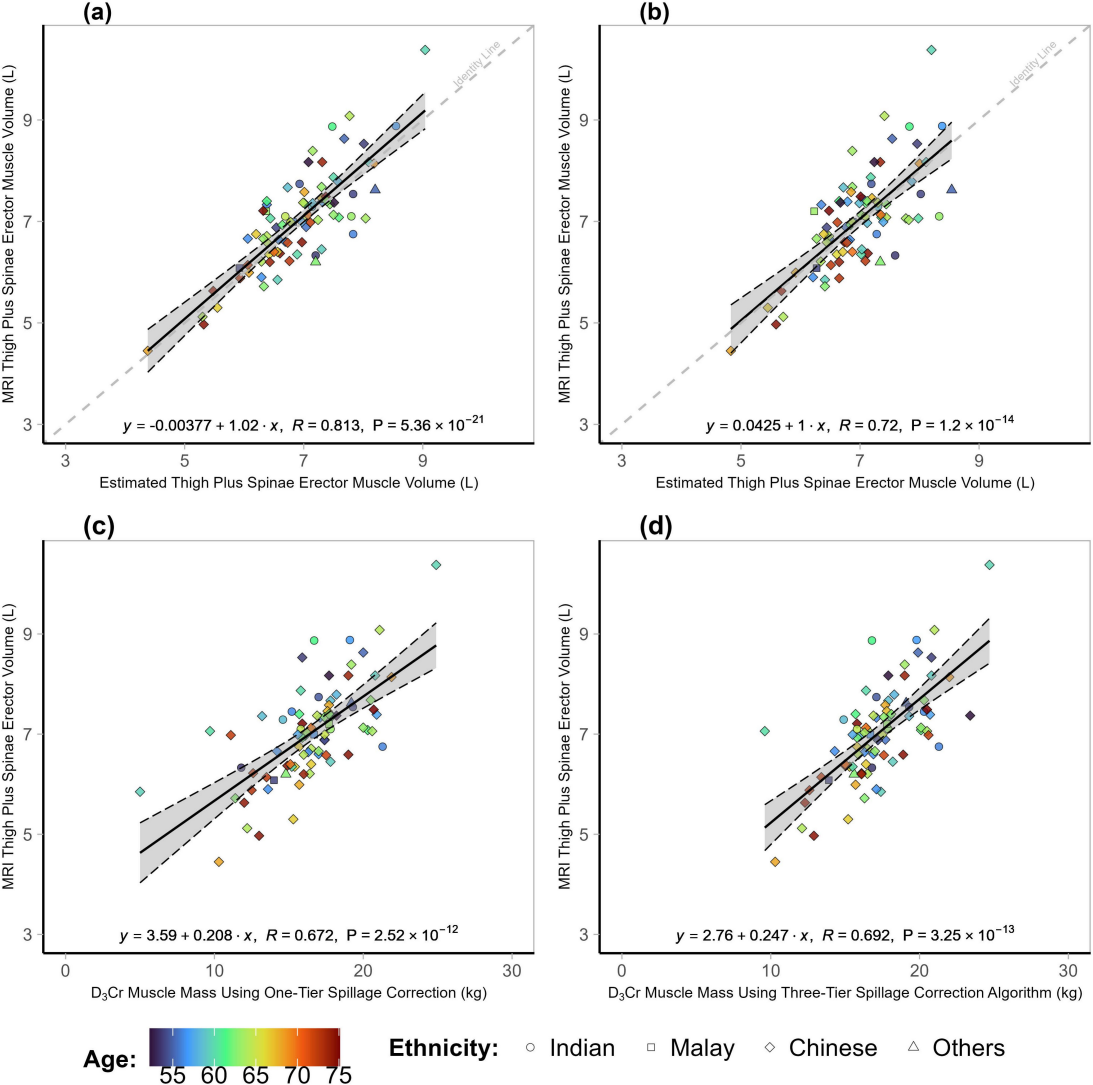
Comparison of MRI‐measured thigh and erector spinae muscle volumes using new versus current algorithms. Upper panels show Pearson correlations of MRI‐measured muscle volumes with estimates using (a) new D_3_Cr_Ht‐Wt_ and (b) MV_Ht‐Wt_ adjusted for height and weight only. Lower panels indicate Pearson correlations of MRI‐measured muscle volumes with total body muscle mass estimates using (c) D_3_Cr_original_ and (d) D_3_Cr_modified_. Individuals (*n* = 84) are from the validation dataset. Regression lines and 95% CI are in grey. Regressions lines for D_3_Cr_original_ and D_3_Cr_modified_ do not intersect zero as they estimate whole‐body muscle mass. Legend: circle = Indian, square = Malay, diamond = Chinese, triangle = others. The heat map indicates age: blue = youngest, red = oldest.

To evaluate the performances of the new D_3_Cr_Ht‐Wt_ and MV_Ht‐Wt_ algorithms, we compared the correlation coefficients of these algorithms to estimate fat‐free muscle volumes in the validation cohort (Figure 3). Estimated muscle mass using the D_3_Cr_Ht‐Wt_ algorithm correlated with MRI‐measured thigh and erector spinae muscle volumes at *R* = 0.813 (Figure 3a). In comparison, correlations using MV_Ht‐Wt_, D_3_Cr_original_ and D_3_Cr_modified_ were *R* = 0.720, *R* = 0.672 and *R* = 0.692, respectively (Figure 3b–d).

Pearson's correlation coefficient of D_3_Cr_Ht‐Wt_ with handgrip strength was 0.411. In comparison, the correlation coefficients of D_3_Cr_original_, D_3_Cr_modified_ and MV_Ht‐Wt_ with handgrip strength were lower at 0.348, 0.389 and 0.339, respectively, in the validation cohort.

**FIGURE 4:**
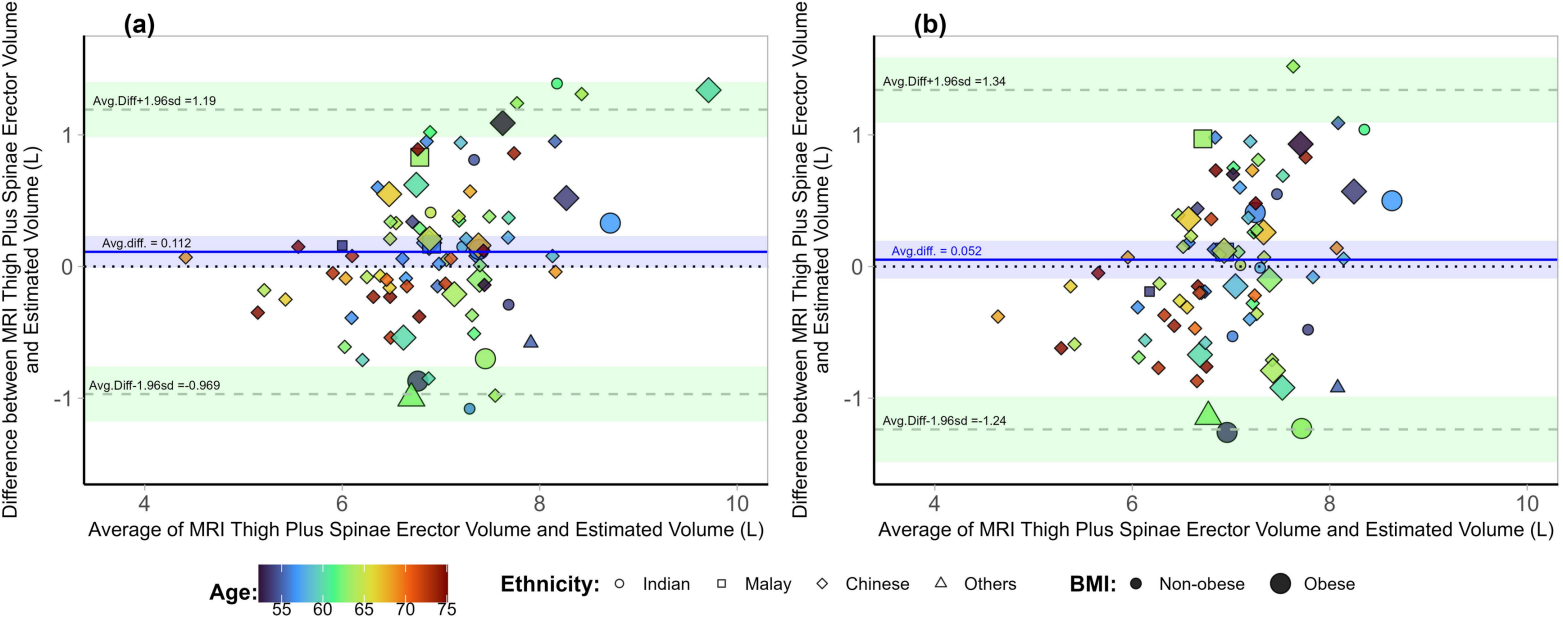
Bland–Altman plots comparing (a) D_3_Cr_Ht‐Wt_ and (b) MV_Ht‐Wt_. D_3_Cr_Ht‐Wt_ had a bias of 0.112 L (blue line) and limits of agreement of −0.969 to + 1.190 L (dotted lines in green regions). MV_Ht‐Wt_ had a bias of 0.052 L (blue line) and limits of agreement of −1.240 to +1.340 L (dotted lines in green regions). The coloured regions represent the 95% confidence intervals around the bias and limits of agreement. Legend: circle = Indian, square = Malay, diamond = Chinese, triangle = others. The heat map indicates age: blue = youngest, red = oldest.

In the Bland–Altman plots, D_3_Cr_Ht‐Wt_ overestimated muscle volume by 0.112 L, with the limits of agreement ranging from −0.969 to +1.190 L (Figure 4a), while MV_Ht‐Wt_ had a lower bias of 0.052 L but wider limits of agreement ranging from −1.240 to 1.340 L (Figure 4b). Both methods displayed good agreement, but narrower limits of agreement in D_3_Cr_Ht‐Wt_ suggested less variability of individual differences.

## 4 Discussion

We report a new D_3_Cr algorithm to estimate fat‐free muscle volumes in women. Fat‐free muscle volumes estimated by the D_3_Cr_Ht‐Wt_ algorithm correlated well with MRI‐measured fat‐free thigh and erector spinae fat‐free muscle volumes at *R* = 0.813, with a low bias and narrow limits of agreement in the Bland–Altman analysis. In comparison, the correlation coefficient using the MV_Ht‐Wt_ algorithm was *R* = 0.720, while the correlation coefficients for D_3_Cr_original_ and D_3_Cr_modified_ were *R* = 0.672 and *R* = 0.692, respectively, in the validation cohort. Age and ethnicity were not significant in our model, suggesting that the proposed algorithm, D_3_Cr_Ht‐Wt_, may be applicable to women of different age ranges and ethnic groups.

Females have 2.5‐fold greater fat content in skeletal muscle than males [30]. Adverse muscle composition is associated with increased risk of poor functional performance, increased metabolic comorbidities and all‐cause mortality [21, 31]. Age‐ and sex‐specific differences also complicate D_3_Cr‐based muscle mass estimation between women and men. Older women may have higher excretion of the D_3_Cr dose compared to men and younger women [10, 14]. Renal excretion of ingested D_3_Cr was lowest in young men, who excrete < 2% of the ingested dose, whereas women excreted the greatest percentage of their dose in urine, ranging from 16.1% in the postmenopausal women to 17.5% in the older women [10]. In the Study of Muscle, Mobility and Aging, involving more than 800 elderly men and women, the correlation of D_3_Cr muscle mass with MRI thigh muscle volume was 0.62 in men and 0.51 in women [28]. The correlation of D_3_Cr muscle mass with objectively measured physical performance was better in men (ranging from 0.28 to 0.40 in men and from 0.13 to 0.19 in women) after adjustment for height and weight [28]. Greater inaccuracies in D_3_Cr muscle mass estimation in women compared to men [10, 11] compel the need for improved algorithms targeted at women.

In addition, the original deuterated creatinine method utilizing one‐tier spillage correction (D_3_Cr_original_) was associated with very low, or even negative, muscle mass in some women in our cohort. The inconsistent performance of the D_3_Cr_original_ algorithm to estimate muscle mass was due to high concentrations of creatine in the urine in some subjects, resulting in abnormally high creatine/creatinine ratios and spuriously high calculated D_3_Cr spillage. In four women in our cohort, calculated D_3_Cr urinary spillage was higher than the 30 mg ingested D_3_Cr dose. Reasons for high creatine levels in urine are unclear. Diets with high creatine intake may increase creatine excretion [32]. The concentration of creatine in muscles can also differ depending on physical fitness [33, 34]. As a result, muscle mass estimated with the D_3_Cr_original_ algorithm had relatively weak correlations with CT and DXA in community‐dwelling post‐menopausal women [16]. These factors might have necessitated the introduction very recently of a modified three‐tier spillage algorithm (D_3_Cr_modified_) for subjects with high urinary creatine levels [28]. However, the biological basis for this modification has yet to be clarified.

Nonetheless, we determined total skeletal muscle mass using D_3_Cr_modified_ for comparison with D_3_Cr_original_. Although negative muscle volumes were no longer observed with the D_3_Cr_modified_ algorithm, its introduction introduces new complexity for sarcopenia studies using the D_3_Cr method. Both D_3_Cr_original_ and D_3_Cr_modified_ rely on empirically developed spillage correction formulae, which might not sufficiently capture the variations in different cohorts. Firstly, the spillage correction varied across age and sex [10, 14]. Secondly, skeletal muscle creatine concentrations were assumed to be consistent at 4.3 g/kg, despite observed variations across different muscle groups [12], age [35], level of physical activity [33, 34] and diet [12, 34]. Difficulties with the current algorithms prompted us to devise a new algorithm that omits the spillage correction. We use regression modelling to identify the factors that were independently associated with MRI‐measured muscle volumes, exploiting the availability of the MRI‐based body composition methodology that can measure fat‐free muscle volumes in the thigh and erector spinae as reference [20, 21, 31]. Since intermuscular and intramuscular fat infiltration has increasingly been recognized to be associated with muscle‐related adverse health outcomes [21, 36], the use of fat‐free muscle volumes as the reference would better clarify the relationships between muscle volumes and important health outcomes.

Our relatively simple D_3_Cr algorithm with height and weight (D_3_Cr_Ht‐Wt_) but omitting the need for spillage correction to estimate fat‐free muscle mass has the potential to facilitate studies on the role of fat‐free muscle volumes in relation to ageing and metabolic disease. Several studies had attempted to develop prediction models for total skeletal mass based solely on anthropometric measurements. Using whole‐body MRI as the reference, Lee et al. developed a model that included skinfold‐corrected upper arm, thigh and calf circumferences and another model that included height and weight [37]. Similarly, Al‐Gindan et al. developed sex‐specific models using height, weight, hip and thigh circumferences [38]. However, models that include only anthropometric features may not be suitable for those who are malnourished or have higher physical fitness and may have low predictive ability in obese individuals [38]. The enrichment ratio, which estimates the turnover of D_3_Cr to D_3_Crn, accounted for metabolic individuality and at the same time serves to discern between fat and muscle, allowing for accurate measurements even in obese individuals.

Current field methods for determining muscle mass, such as DXA or BIA, correlated poorly with functional capacity and health‐related outcomes, leading to the conclusion that the amount of muscle may not be associated with these age‐associated outcomes [5]. The D_3_Cr_Ht‐Wt_ algorithm was anchored on the fundamental role of creatine metabolism in the myofibril contractile apparatus, thus removing the women‐specific problematic spillage correction and incorporating easily measured height and weight parameters. It has the potential for community‐wide screening, which will have clinical and research relevance, particularly for sarcopenia studies in midlife women. A challenge for the future is to establish the validity of the D_3_Cr_Ht‐Wt_ algorithm in different populations and over different clinical settings.

Our study has several limitations. The linear equation was developed based on an Asian midlife woman and may not be generalizable to other populations. However, ethnicity was not significant in our regression analyses, and there is no evidence indicating that the biology of creatine metabolism differs between ethnicities [12]. The D_3_Cr_Ht‐Wt_ algorithm also estimated thigh and spinae erector muscle volumes, not whole‐body muscle volumes. Nevertheless, since the proportion of thigh and erector spinae muscles to whole‐body skeletal muscle mass of ~45% [25] is relatively consistent for healthy individuals and have a high correlation (*R* ^2^ = 0.90, *p* < 0.05) with whole‐body and thigh skeletal muscle volumes in women [39], whole‐body muscle volume, if required, can be derived by the following formula: D_3_Cr_Ht‐Wt_ (L)/0.45. The method also depends on the willingness of women to orally ingest a small dose D_3_Cr and be tested in a fasted state. The D_3_Cr enrichment ratio calculations assume that the amount of D_3_Cr outside of skeletal muscle is largely negligible and that this amount is stable with respect to diet, exercise or training status.

A strength of our study is the relatively large number of subjects. Another strength is the use of gold‐standard MRI‐measured fat‐free muscle mass as reference. Intermuscular adipose tissue has been extensively implicated in metabolic diseases such as insulin resistance and Type 2 diabetes mellitus [36]. Intramuscular adipose tissue is negatively correlated with muscle strength and performance and is representative of early architectural changes in musculature prior to the loss of strength, mobility and metabolic conditions with age [40]. The D_3_Cr method is independent of changes in renal excretion of creatinine as the ratio of D_3_Crn to endogenous creatinine was used to calculate muscle mass rather than absolute values. The D_3_Cr_Ht‐Wt_ algorithm also does not depend on the measurement of urinary creatine, which may be affected by renal function. Finally, the recent availability of reference ranges for MRI‐measured fat‐free muscle mass parameters in a large community‐dwelling population coupled with data on frailty, falls and mortality [15, 18, 20, 21, 31] would facilitate the utility of the D_3_Cr_Ht‐Wt_ algorithm for sarcopenia studies in the community.

In summary, our new D_3_Cr_Ht‐Wt_ algorithm, incorporating height and weight but omitting spillage correction, correlated well with gold‐standard MRI‐measured fat‐free thigh and erector spinae muscle volumes. Given the feasibility of the D_3_Cr method for community studies, it has the potential to become a clinically relevant method for assessing skeletal muscle volumes in sarcopenia studies if validated in other populations. More studies need to be conducted to explore the associations between fat‐free muscle volumes estimated by the D_3_Cr_Ht‐Wt_ algorithm with clinically relevant outcomes related to muscle function.

## Conflicts of Interest

The authors declare no conflicts of interest.

## Supporting information


**Figure S1.** Chromatograms and product ion spectra (close‐up panel on the right) of D_3_‐creatinine, creatinine, creatine and deuterated‐labelled internal standards, D_5_‐creatinine and D_5_‐creatine. The part of the molecule shaded in red is the distinctive product ion yield after collisional dissociation and was selected as the MRM transition.

**Table S1.** Regression analysis of calibration curves of each analyte in human urine.
**Table S2.** Validation summary of the equivalency of creatinine versus D_3_‐creatinine.
**Table S3.** Creatinine response ratio (M + 2)/(M + 0) determined using LC–MS/MS. Peak area ratio was determined using different creatinine standard concentrations, 500 ng/mL for M + 2 and 10 ng/mL for M + 0.
**Table S4.** Validation of in‐house corrective factor, 0.002469, using creatinine (measured as M + 2) and D_3_‐creatinine standards, both at a concentration of 200 ng/mL.
**Table S5**. Validation summary of quality control (QC) mean, standard deviation, precision and bias statistics including between‐run precision of the analytes, D_3_‐creatinine, D_5_‐creatinine and D_5_‐creatine, at four different concentration levels, in human urine matrix.
**Table S6.** Characteristics of participants who were included in (*n* = 451) and excluded from (*n* = 443) this analysis. Continuous variables are reported as mean ± SD, and categorical variables are reported as *n* (%).
**Table S7**. Simple correlation analysis of key variables in the model development group with MRI‐measured fat‐free thigh and erector spinae muscle volumes.
