# Supplementary figures and images for: Novel Algorithm to Estimate Fat‐Free Muscle Volumes in Women Using the Urinary Deuterated‐Creatine Dilution Method
Source: J Cachexia Sarcopenia Muscle. 2025 Jul 9;16(4):e13872. doi: 10.1002/jcsm.13872 (PMC12238901; doi:10.1002/jcsm.13872)

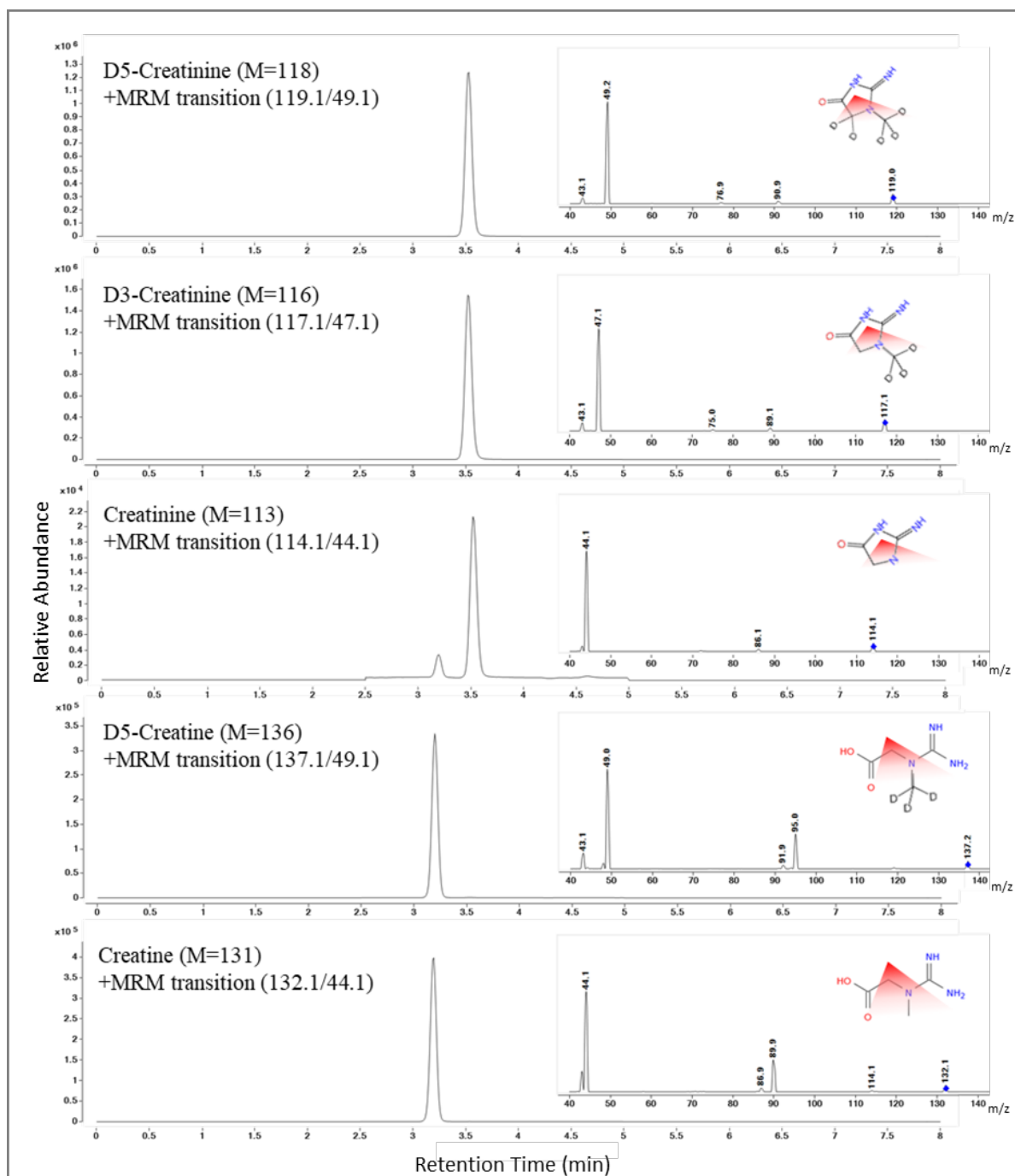

Supplement: Supplementary file 1 — Figure S1. Chromatograms and product ion spectra (close‐up panel on the right) of D3‐creatinine, creatinine, creatine and deuterated‐labelled internal standards, D5‐creatinine and D5‐creatine. The part of the molecule shaded in red is the distinctive product ion yield after collisional dissociation and was selected as the MRM transition. [file JCSM-16-e13872-s002.pdf]
