# Supplementary material for: Novel Algorithm to Estimate Fat‐Free Muscle Volumes in Women Using the Urinary Deuterated‐Creatine Dilution Method
Source: J Cachexia Sarcopenia Muscle. 2025 Jul 9;16(4):e13872. doi: 10.1002/jcsm.13872 (PMC12238901; doi:10.1002/jcsm.13872)
Supplement: Supplementary file 2 — Table S1. Regression analysis of calibration curves of each analyte in human urine. Table S2. Validation summary of the equivalency of creatinine versus D3‐creatinine. Table S3. Creatinine response ratio (M + 2)/(M + 0) determined using LC–MS/MS. Peak area ratio was determined using different creatinine standard concentrations, 500 ng/mL for M + 2 and 10 ng/mL for M + 0. Table S4. Validation of in‐house corrective factor, 0.002469, using creatinine (measured as M + 2) and D3‐creatinine standards, both at a concentration of 200 ng/mL. Table S5. Validation summary of quality control (QC) mean, standard deviation, precision and bias statistics including between‐run precision of the analytes, D3‐creatinine, D5‐creatinine and D5‐creatine, at four different concentration levels, in human urine matrix. Table S6. Characteristics of participants who were included in (n = 451) and excluded from (n = 443) this analysis. Continuous variables are reported as mean ± SD, and categorical variables are reported as n (%). Table S7. Simple correlation analysis of key variables in the model development group with MRI‐measured fat‐free thigh and erector spinae muscle volumes. [file JCSM-16-e13872-s001.pdf]

TITLE PAGE

Novel algorithm to estimate fat-free muscle volumes in women using the urinary deuterated-creatine dilution method

Darren Yuen Zhang Tan[1*], Wei Fun Cheong[1*], Shanshan Ji[2], Amaury Cazenave-Gassiot[2,3], Jane Cauley[4], Liang Shen[5], and Eu-Leong Yong[1]

[1]Department of Obstetrics and Gynecology, Yong Loo Lin School of Medicine, National University of Singapore, NUHS Tower Block, 1E Kent Ridge Road, Level 12, Singapore, 119228, Republic of Singapore.

[2]Singapore Lipidomics Incubator, Life Sciences Institute, National University of Singapore, Singapore, 117456, Republic of Singapore.

[3]Department of Biochemistry, Yong Loo Lin School of Medicine, National University of Singapore, Singapore, 117596, Republic of Singapore.

[4]Department of Epidemiology, University of Pittsburgh, Graduate School of Public Health, Pittsburgh, PA, USA.

[5]Biostatistics Unit, Yong Loo Lin School of Medicine, National University of Singapore, Singapore, 117597, Republic of Singapore.

**Co-first authors.*

Corresponding author:
Eu-Leong Yong, FRCOG, PhD
obgyel@nus.edu.sg
Tel: +65 6772 4278

Table S1. Regression analysis of calibration curves of each analyte in human urine.

Standard	Precursor Ion (m/z)	Product Ion (m/z)	Fragmentor (V)	Collision Energy (V)	Linearity (ng/mL)	LOD (ng/mL)	LOQ (ng/mL)	*R2*	Equation
**D_3-creatinine** (1,2,2-D_3)	117.1	47.1	380	16	0.1 - 250	0.55	1.83	1	$y = 9519.9x - 733.55$
					0.1 - 5	0.11	0.38	0.9996	$y = 9316.6x -36.982$
**D_5-creatinine (*IS*)** (26,26,26,27,27-D_5)	119.1	49.1	380	16	0.1 - 250	0.99	3.29	1	$y = 9072.8x - 1655.2$
					0.1 - 50	0.17	0.58	1	$y = 8917.8x - 69.003$
**D_5-creatine (*IS*)** (23,24,25,26,27-D_5)	137.1	49.1	380	16	0.1 - 10,000	N/A	N/A	0.9968	$y = -0.4012x^2 + 8180.2x + 365618$
					0.1 - 2,500	N/A	N/A	1	$y = -0.8824x^2 + 10244x + 12995$
					0.1 - 500	13.07	43.56	0.9997	$y = 9788.2x + 23874$

Table S2. Validation summary of the equivalency of creatinine versus D_3-creatinine.

**Standard concentration (ng/mL)**	**Creatinine (M+0)** Peak area	**D_3-creatinine (M+0)** Peak area	**D_3-creatinine vs creatinine** (%)	**D_3-creatinine vs creatinine** (% difference)
200	4,698,588	4,646,520	101.1	1.1
200	4,706,347	4,662,803	100.9	0.9
200	4,730,758	4,579,300	103.3	3.3
200	4,691,459	4,686,030	100.1	0.1
200	4,708,208	4,699,915	100.2	0.2
200	4,741,302	4,724,261	100.4	0.4
200	4,701,373	4,694,716	100.1	0.1
200	4,701,538	4,589,438	102.4	2.4
200	4,611,978	4,647,729	99.2	-0.8
200	4,528,993	4,611,381	98.2	-1.8
200	4,590,621	4,620,250	99.4	-0.6
200	4,513,942	4,701,651	96.0	-4.0
Mean			***100.1***	***0.1***

Table S3. Creatinine response ratio (M+2)/(M+0) determined using LC-MS/MS. Peak area ratio was determined using different creatinine standard concentrations, 500ng/mL for M+2 and 10ng/mL for M+0.

Peak area ratio			
**Creatinine (M+2) 500ng/mL (116.1/44) [A]**	**Creatinine (M+0) 10ng/mL (114.1/44) [B]**	**Creatinine (M+0) corrected[1] [B] x 50 (dilution factor) = [C]**	**Response ratio [A] / [C]**
0.004357	0.034892	1.74	0.002497
0.004154	0.034627	1.73	0.002399
0.004317	0.033817	1.69	0.002553
0.004232	0.034198	1.71	0.002475
0.004231	0.033184	1.66	0.002550
0.004381	0.034935	1.75	0.002508
0.004325	0.035306	1.77	0.002450
0.004313	0.034700	1.73	0.002486
0.004351	0.035304	1.77	0.002465
0.004342	0.034755	1.74	0.002499
0.004248	0.034934	1.75	0.002432
0.004190	0.036191	1.81	0.002316
Mean			
0.004287	*0.034737*	*1.736839*	***0.002469***
%RSD			
1.7	*2.2*	*2.2*	*2.7*

[1] *Corrected for concentration difference.*

Table S4. Validation of in-house corrective factor, 0.002469, using creatinine (measured as M+2) and D_3-creatinine standards, both at 200ng/mL concentration.

Standard concentration (ng/mL)	Peak area			D_3-creatinine vs creatinine (%)
	Creatinine (M+2) (MRM 116.1/44)	Creatinine M+2[1] corrected as to M+0	D_3-creatinine (MRM 117.1/47)	
200	11,534	4,670,937	4,646,520	99.5
200	11,331	4,588,923	4,662,803	101.6
200	11,611	4,702,032	4,579,300	97.4
200	11,318	4,583,385	4,686,030	102.2
200	11,325	4,586,385	4,699,915	102.5
200	11,169	4,522,975	4,724,261	104.5
200	11,017	4,461,430	4,694,716	105.2
200	11,387	4,611,426	4,589,438	99.5
200	11,074	4,484,773	4,647,729	103.6
200	11,337	4,591,098	4,611,381	100.4
200	11,120	4,503,339	4,620,250	102.6
200	11,394	4,614,202	4,701,651	101.9
Mean	***11,301***	***4,576,742***	***4,655,333***	***101.8***
%RSD	***1.6***	***1.6***	***1.0***	***2.2***

[1] *Corrected peak area (divided by mean response ratio of 0.002469).*

Table S5. Validation summary of quality control (QC) mean, standard deviation, precision, and bias statistics including between-run precision of the analytes, D_3-creatinine, D_5-creatinine, and D_5-creatine, at 4 different concentration levels, in human urine matrix.

	**Nominal concentration of *D_3-creatinine* in human urine matrix (QC)**			
	**50ng/mL**	**500ng/mL**	**3000ng/mL**	**5000ng/mL**
Mean of Measure Conc. (ng/mL)	48.5	485.6	3105.9	4300.3
SD	3.1	25.9	68.9	335.4
Precision (%)	1.4	1.2	1.0	0.5
Bias (%)	96.9	97.1	103.5	86.0
n	5	5	5	5
Between-run precision (%)	7.1	6.1	2.5	1.3
	**Nominal concentration of *D_5-creatinine* in human urine matrix (QC)**			
	**50ng/mL**	**500ng/mL**	**3000ng/mL**	**5000ng/mL**
Mean of Measure Conc. (ng/mL)	51.8	523.6	3290.0	4707.4
SD	4.9	61.7	176.9	133.9
Precision (%)	0.9	1.4	0.7	0.5
Bias (%)	103.6	104.7	109.7	94.1
n	5	5	5	5
Between-run precision (%)	4.1	5.9	5.4	2.8
	**Nominal concentration of *D_5-creatine* in human urine matrix (QC)**			
	**50ng/mL**	**500ng/mL**	**3000ng/mL**	**5000ng/mL**
Mean of Measure Conc. (ng/mL)	50.2	461.6	2717.3	4270.2
SD	3.0	24.6	68.4	566.6
Precision (%)	2.0	1.5	0.8	0.8
Bias (%)	100.3	92.3	90.6	85.4
n	5	5	5	5
Between-run precision (%)	6.0	5.3	2.5	3.9

Table S6. Characteristics of participants who were included in (n = 451) and excluded (n = 443) from this analysis. Continuous variables are reported as mean ± SD and categorical variables are reported as n (%).

	Participants re-contacted (n = 894)	Included in analysis (n = 451)	Excluded from analysis (n = 443)	p value
Age	62.8 ± 6.0	62.6 ± 5.9	63.0 ± 6.2	0.327
Ethnicity				0.109
Chinese	725 (81.1)	368 (50.8)	357 (49.2)	
Malay	50 (5.6)	27 (54.0)	23 (46.0)	
Indian	90 (10.1)	37 (41.1)	53 (58.9)	
Other	29 (3.2)	19 (65.5)	10 (34.5)	
Education				**<0.001**
No formal/primary	105 (11.9)	43 (41.0)	62 (59.0)	
Secondary and Pre-University	584 (66.4)	286 (49.0)	298 (51.0)	
University	191 (21.7)	118 (61.8)	73 (38.2)	
Monthly Household Income				0.058
<$3,000	201 (29.2)	92 (45.8)	109 (54.2)	
$3,000-$6,999	223 (32.4)	121 (54.3)	102 (45.7)	
≥$7,000	265 (38.5)	150 (56.6)	115 (43.4)	
Marital Status				0.475
Not Married	203 (22.8)	107 (52.7)	96 (47.3)	
Married	690 (77.3)	344 (49.9)	346 (50.1)	
Employment Status				0.980
Unemployed	377 (42.2)	190 (50.4)	187 (49.6)	
Employed	517 (57.8)	261 (50.5)	256 (49.5)	
Menopausal Status				0.622
Pre-menopausal	9 (1.0)	6 (66.7)	3 (33.3)	
Peri-menopausal	22 (2.5)	11 (50.0)	11 (50.0)	
Post-menopausal	852 (96.5)	429 (50.4)	423 (49.6)	
Height (m)	1.56 ± 0.06	1.56 ± 0.06	1.56 ± 0.06	0.477
Weight (kg)	59.5 ± 11.2	59.5 ± 10.8	59.5 ± 11.6	0.967
BMI (kg/m^2)	24.6 ± 4.6	24.6 ± 4.4	24.6 ± 4.8	0.834

Table S7. Simple correlation analysis of key variables in model development group with MRI-measured fat-free thigh and erector spinae muscle volumes.

Variables	*r*	*p* value
Age	-0.280	<0.001
Height	0.475	<0.001
Weight	0.566	<0.001
BMI	0.373	<0.001
Enrichment ratio	-0.628	<0.001
